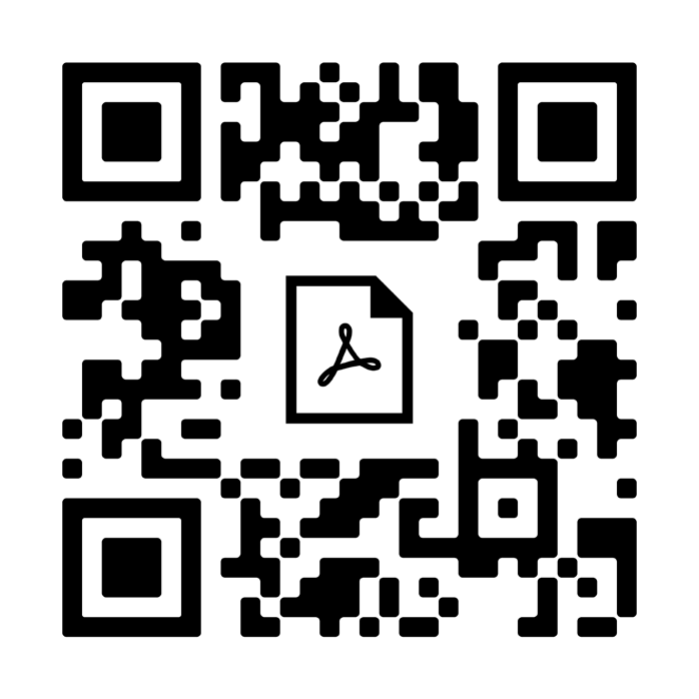# Practice of laparoscopic prolapse surgery in Europe - ESGE Survey

**DOI:** 10.52054/FVVO.15.3.087

**Published:** 2023-09-24

**Authors:** B Lambert, L de Landsheere, GK Noé, R Devassy, H Ferreira, J duBuisson, J Deprest, R Botchorishvili

**Affiliations:** Department of Obstetrics and Gynecology, University of Liège, CHR Citadelle, 4000 Liège, Belgium; Department of Obstetrics and Gynecology, University of Witten-Herdercke, 41540 Dormagen, Germany; Department of Obstetrics and Gynecology, Dubai London Clinic and Speciality Hospital, 12119 Dubai, United Arab Emirates; Department of Minimally Invasive Surgery Unit of Centro Hospitalar Universitário do Porto, 4099 Porto, 12 Portugal; Department of Obstetrics and Gynecology, University Hospitals of Geneva, 1205, Geneva, Switzerland; Pelvic Floor Unit, University Hospitals Leuven, and Cluster Urogenital Surgery, Department Development and Regeneration, Biomedical sciences, KU Leuven, 3000 Leuven, Belgium; Department of Gynecological Surgery, University Hospital Estaing, 63000 Clermont-Ferrand, France

**Keywords:** Prolapse surgery, sacrocolpopexy, pectopexy, lateral suspension, meshless laparoscopic repair, survey

## Abstract

Sacrocolpopexy is considered as the “gold standard” for management of women with apical prolapse. Numerous technical variants are being practiced. The first aim of this survey was to determine the habits of practice of laparoscopic sacrocolpopexy (LSCP) in Europe. The second aim was to determine whether surgeons who perform laparoscopic pelvic organ prolapse (POP) repair are familiar with the practice of alternative techniques and with mesh-less laparoscopic treatment of prolapse. The questionnaire was designed by the Urogynaecology Special Interest Group of the European Society for Gynaecological Endoscopy (ESGE). All ESGE-members were invited by email to respond to this survey consisting of 54 questions divided in different categories. Following review of ESGE member’s responses, we have highlighted the great heterogeneity concerning the practice of LSCP and important variability in performance of concomitant surgeries. Alternative techniques are rarely used in practice. Furthermore, the lack of standardisation of the many surgical steps of a laparoscopic sacrocolpopexy is mainly due to the lack of evidence. There is a need for training and teaching in both standard and newer innovative techniques as well as the reporting of medium and long-term outcomes of both standard laparoscopic sacrocolpopexy and any of its alternatives.

## Introduction

Pelvic organ prolapse (POP) is a highly prevalent condition requiring surgical treatment in 6-18% of cases (Barber and Maher, 2013). Different surgical approaches are described for the treatment of apical defects with sacrocolpopexy still considered to be the gold standard (Maher et al., 2013). Because of the recent events related to transvaginal mesh banning in Europe, laparoscopic management of POP has evolved. Alternative techniques such as pectopexy or lateral suspension (LS) allow the management of POP in case of anatomical variations. Laparoscopic pectopexy (LP) consists of bilateral suspension with mesh using the iliopectineal (Cooper’s) ligament. Lateral suspension was described for the first time by Kapandji in 1968 and the technique has been modified and updated for a laparoscopic approach by Dubuisson in 1998 (Mereu et al., 2018). This alternative procedure is especially indicated for apical and anterior POP repair whereby the mesh is fixed to the vagina or cervix, with two mesh arms connected laterally to the abdominal wall after creating an extraperitoneal tunnel toward the round ligament (Dubuisson et al., 2008; Simoncini et al., 2016; Veit-Rubin et al., 2017).

The possible extension of restriction to mesh use in abdominal repair of prolapse, or even stress urinary incontinence, reinforces the need for developing mesh-less laparoscopic treatments for these conditions. These techniques may present a valuable alternative to the usual laparoscopic surgeries with good results in clinical studies (Syed et al., 2021). The aim of this survey is to determine the habits of practice of laparoscopic sacrocolpopexy (LSCP) in Europe and whether surgeons who perform laparoscopic pelvic floor reconstruction are familiar with the practice of these alternative techniques.

## Materials and Methods

The questionnaire has been designed by the Urogynaecology Special Interest Group of the ESGE. The questionnaire consisted of 54 questions divided in different categories: demographics and surgeon characteristics, concomitant hysterectomy, and characteristics of surgery. Finally, surgeons were questioned about attitude towards practice of pectopexy, LS and laparoscopic native tissue repair. All the members of the ESGE were invited by email to respond to this survey. Respondents were allowed to skip any questions they wished. For statistical analysis, results were presented as an absolute number and percentage of the respondents answering the question.

## Results

### Demographics

119 surgeons among ESGE members responded to the questionnaire. Most of them (84.2%) had over 5 years of experience in laparoscopic procedures. Only 36.8% of the physicians dedicated over 50% of their work activity to urogynaecology. In total, 61.4% reported having a specific training in pelvic floor reconstructive surgery. Since the withdrawal of transvaginal mesh, 48.8% of the surgeons had no change in number of laparoscopic prolapse surgeries whereas 41.5% increased their number of laparoscopic POP repair. Among the multiple types of laparoscopic prolapse surgeries, 84.2% of the respondents answered perform sacrocolpopexy, 29.3% were accustomed with uterosacral ligament plication, 23.2% used native-tissue repair, 20.7% performed LS and only 13.4% used pectopexy.

### Characteristics of prolapse surgery

The characteristics of prolapse surgery are listed in the Table I. Less than 10% of the surgeons sometimes performed these procedures with a robot-assisted approach. Indications for robot-assisted laparoscopy were obesity or recurrence of prolapse. The main cause for its low use were cost issues. Preoperative bowel preparation was not required for 63.4% of the respondents. 95.1% of the physicians recommended routine administration of antibiotics perioperatively. Suspension of the colon with temporary sutures or other devices through appendices epiploic was performed by 53.8% of the respondents only in case of poor visibility while one in four always did and 21.3% never. Most of the surgeons used uterine manipulation (83.8%). Vaginal valves for vaginal exposition were used by 71.3% of the respondents. Various devices were used by the surgeons for cutting and dissection. Overall, 79.5% of physicians dissected the entire peritoneum from the promontory to the Douglas pouch whereas 16.7% of them only performed tunnelling of the caudal peritoneum. Regarding posterior mesh in LSCP, 50% dissected the rectovaginal space and the levator ani muscles on both sides. On the anterior mesh, most respondents continued their dissection of the vesico-uterine space as deep as possible (46.2%).

**Table I:** Characteristics of prolapse surgery.

		N	%
Team composition in the operating room (multiple answers allowed)			
	Senior gynaecologist with assistant(s)	72/81	88.89
	Two senior gynaecologists	13/81	16.05
	Senior gynaecologist with a urologist	3/81	3.70
	Senior gynaecologist with a general surgeon	3/81	3.70
Scrub nurse presence			
	Always	65/82	79.27
	Never	9/82	10.98
Robot-assisted approach			
	No	74/82	90.24
	Always	0/82	0.00
	Sometimes	8/82	9.76
Preoperative bowel preparation			
	Yes	30/82	36.59
	No	52/82	63.41
Routine administration of antibiotics perioperatively			
	Yes	78/82	95.12
	No	4/82	4.88
Routine administration of antibiotics postoperatively			
	Yes	21/82	25.61
	No	61/82	74.39
Suspension of colon			
	Yes, always.	20/80	25.00
	No, never.	17/80	21.25
	Only in case of poor visibility	43/80	53.75
	Other	0/80	0.0
Uterine manipulator use			
	Yes	67/80	83.75
	No	13/80	16.25
Vaginal valves use for vaginal exposition.			
	Yes	57/80	71.25
	No	23/80	28.75
	Other	0/80	0.00
Device used for cutting and dissection (multiple answers allowed)			
	Scissors	33/78	42.31
	Scissors with monopolar energy mode	40/78	51.28
	Ultrasonic energy	27/78	34.62
	Monopolar hook	17/78	21.79
	Other	9/78	11.54
Dissection of the peritoneum			
	Entire (from the promontory to the Douglas pouch)	62/78	79.49
	Tunnelling of the caudal peritoneum	13/78	16.67
	Other	3/78	33.85
Extension of posterior dissection (multiple answers allowed)			
	Up to the level of cervix/vault and maximally the upper part of the posterior vaginal wall	30/78	38.46
	Up to halfway up the posterior vaginal wall	16/78	20.51
	Dissection of the levator ani muscles on both sides.	39/78	50.00
	Depending on the situation	6/78	7.69
Extension of anterior dissection (multiple answers allowed)			
	Up to the level of the cervix/vault and maximum 1-2 cm of the anterior vaginal wall	22/78	28.21
	Up to 1-2 cm under the bladder	22/78	28.21
	As deep as possible	36/78	46.15
	Depending on the situation	6/78	7.69
Mesh attachment			
	Only posterior	1/75	1.33
	Only anterior	6/75	8.00
	Both anterior and posterior	49/75	65.33
	Depending on the situation	18/75	24.00
	Other	1/75	1.33
Mesh characteristics (multiple answers allowed)			
	Polypropylene mesh	63/74	85.14
	Mersilene mesh	5/74	6.76
	Parietex mesh	2/74	2.70
	Other	11/74	14.86
Mesh shape (multiple answers allowed)			
	Prefabricated Y-shaped mesh	29/74	39.19
	Y-shaped mesh prepared during surgery	27/74	36.49
	Single leaf	7/74	9.46
	Individually cut to fit/adjusted.	32/74	43.24
	Cross like mesh	3/74	4.05
	Other	2/74	2.70
Technique for fixing mesh to the cervix/vault (multiple answers allowed)			
	Absorbable monofilament (PDS or similar)	22/74	29.73
	Absorbable multifilament (VICRYL, POLYSORB or similar)	14/74	18.92
	Non-absorbable monofilament (PROLENE, SURGIPRO, GORE-TEX or similar)	20/74	27.03
	Non-absorbable multifilament (ETHIBOND, MERSUTURE or similar)	34/74	45.95
	Staples	4/74	5.41
	Tackers	6/74	8.11
	Glue	1/74	1.35
	Other	3/74	4.05
Technique for fixing mesh to the levator ani muscles (multiple answers allowed)			
	Absorbable monofilament (PDS or similar)	14/74	18.92
	Absorbable multifilament (VICRYL, POLYSORB or similar)	16/74	21.62
	Non-absorbable monofilament (PROLENE, SURGIPRO, GORE-TEX or similar)	8/74	10.81
	Non-absorbable multifilament (ETHIBOND, MERSUTURE or similar)	16/74	21.62
	Staples	3/74	4.05
	Tackers	3/74	4.05
	Glue	1/74	2.70
	Never attached	16/74	21.62
	Other	3/74	4.05
Attachment of the mesh to the promontory			
	Absorbable monofilament (PDS or similar)	5/74	6.76
	Absorbable multifilament (VICRYL, POLYSORB or similar)	3/74	4.05
	Non-absorbable monofilament (PROLENE, SURGIPRO, GORE-TEX or similar)	14/74	18.92
	Non-absorbable multifilament (ETHIBOND, MERSUTURE or similar)	27/74	36.49
	Staples	4/74	5.41
	Tackers	19/74	25.68
	Glue	0/74	0.00
	Other	2/74	2.70
Number of stitches (or staples, tackers) at the promontory			
	1	23/74	31.08
	2	30/74	40.54
	>3	21/74	28.38

Concerning mesh fixation, 65.3% of respondents attached both anterior and posterior mesh up to the promontory, while 24% did so depending on the situation. The most commonly used type of mesh was polypropylene mesh. Anterior mesh was fixed to the cervix/vagina with both absorbable and non- absorbable sutures. Posterior mesh was never fixed for 21.6% of the respondents, whereas absorbable and non-absorbable sutures were mostly used to fix the mesh to the levator ani muscles. The mesh was fixed to the promontory with non-absorbable sutures by 55.4% and with tackers by 25.7%. Overall, 62.2% of the surgeons fixed anterior and posterior meshes to the promontory, while 27% fixed only the anterior mesh. 41.9% of the surgeons apply tension free and 37.8% slight tension to fix the mesh to the promontory. For most surgeons (56.8%), the tension was assessed both visually, by laparoscopy, and by vaginal examination. All the surgeons closed the peritoneum to cover the mesh. 79.7% of them used absorbable sutures and 16.2% used a self-locking barbed suture. Overall, after laparoscopic prolapse surgery, the duration of stay at the hospital was one day for 29.3% of the respondents, two days for 50.7%, more than two days for 16%, while the procedure was carried out in day surgery for 4%.

### Concomitant surgery (Table II)

Nearly half of the respondents preferred performing laparoscopic supracervical hysterectomy before prolapse repair. 60.8% of the surgeons never performed concomitant surgery for stress urinary incontinence (SUI), whereas 10.8% and 28.4% did it routinely and depending on the situation respectively. Most of these used a sub-urethral sling preferentially.

**Table I:** Concomitant surgery.

		N	%
Supracervical hysterectomy during pelvic organ prolapse repair			
	Yes	45/98	45.92
	No, preference for hysteropexy	26/98	26.53
	No, only sacrocolpopexy in cases of post-hysterectomy vaginal vault prolapse	20/98	20.41
	Sometimes	7/98	7.14
Total hysterectomy accepted doing mesh prolapse surgery by laparoscopy			
	Yes	25/98	25.51
	No	64/98	65.31
	Sometimes	9/98	9.18
Concomitant colporrhaphy			
	Before laparoscopy	8/74	10.81
	After laparoscopy	25/74	33.78
	No	34/74	45.95
	Other	7/74	9.46
Concomitant surgery for SUI			
	Systematically	8/74	10.81
	Never	45/74	60.81
	Depending on the situation	21/74	28.38
Technique of concomitant surgery for SUI			
	Never performed	30/73	41.10
	Retropubic TVT	7/73	9.59
	TVT-O	15/73	20.55
	TOT	14/73	19.18
	Single incision sling	5/73	6.85
	Burch colposuspension	11/73	15.07
	Bulkamid	3/73	4.11
	Other	1/73	1.37

### Practice of pectopexy

Laparoscopic pectopexy was performed by 16% of the respondents. The indication for pectopexy was difficult access to promontory for 63.6%, while for the remaining 36.4% this was routinely done.

### Practice of lateral suspension

Laparoscopic lateral suspension was performed more often than pectopexy (28.4%). In 13.6% of the respondents, this procedure represented more than 90% of their activity. The principal indications remained difficult access to the promontory and obesity. In 30% of the respondents, this surgery was routinely done instead of LSCP. In the same way as for pectopexy, only mesh suspension points changed. Other main steps stayed the same as for LSCP.

### Practice of laparoscopic native tissue repair

Laparoscopic native tissue repair was performed by 25.7% of the respondents. Different indications were cited by the respondents, but the main reason was the risk of mesh-related complication. Various approaches to the laparoscopic native tissue repair exist: anterior and posterior colporrhaphy, utero- sacral ligament plication and suspension of the vaginal vault/cervix to the promontory with suture.

## Discussion

This survey provides an insight into the habits of practice of LSCP among ESGE members who perform laparoscopic pelvic floor reconstruction and their familiarity with the practice of alternative techniques such as pectopexy, LS or native tissue repair. We have found that there is a wide heterogeneity in techniques for LSCP and marked variations in the recommendations found in recent literature.

The extent of dissection of the vesico-vaginal space varies between authors (Moroni et al., 2018). There is a consensus based on various publications on the positioning and fixation of the anterior prosthesis: the anterior mesh should be attached to the upper part of the uterine isthmus and to the anterior surface of the vagina for the lower part (Ganatra et al., 2009). However, with regards to the extent of dissection within the recto-vaginal space, there is currently no consensus in the literature. Although fixation of the mesh to the levator ani has been described, the latest reviews tend to prefer fixation of the mesh to the posterior vagina alone (Moroni et al., 2018). These differences reflect the lack of clear guidance in the literature. Variations may arise due to the diversity in patient groups, demonstrating that one technique does not fit all.

A prophylactic posterior mesh is often used during LSCP to prevent de novo posterior prolapse, although there is a lack of evidence for this practice. The LAparoscopic Preventative PRe- Rectal Mesh (LAPREM) trial, which is currently being conducted by Lucot and colleagues is trying to answer this question through a randomised, double-blinded, non-inferiority-controlled trial comparing the results of LSCP with or without use of preventive pre-rectal mesh in women admitted for urogenital prolapse (without significant posterior vaginal wall prolapse). One retrospective study compared absorbable and permanent sutures for vaginal mesh attachment and found no difference in recurrence rate. The main fear regarding the use of non-absorbable sutures on the vagina is the possible risk of mesh or suture erosion (Tan-Kim et al., 2014).

Presently, there is no consensus regarding the best way to fix the mesh to the promontory, as non-absorbable sutures, staples, and tackers have all proven to have similar results (Costantini et al., 2016; Moroni et al., 2018). In the absence of comparative studies, it is the opinion of the authors that fixation to the promontory with non-absorbable sutures should be preferentially used to avoid the risk of spondylodiscitis, which has occasionally been described with the use of tackers.

All authors recommend a systematic peritonealisation of the meshes (Costantini et al., 2016; Ganatra et al., 2009; Moroni et al., 2018). Most appropriate type of mesh and suture remains controversial, but currently type I monofilament polypropylene meshes (Amid Classification) are recommended.

Before surgical management of POP, the decision must be made whether to perform concomitant surgery such as hysterectomy, or surgery for SUI, as a part of the procedure. Although there is no difference with or without hysterectomy in term of results, the role of uterine preservation versus hysterectomy and colpopexy is controversial, as the main concern with concomitant total hysterectomy is a higher risk of mesh exposure (Costantini et al., 2016). The authors do not advocate the use of mesh when a total hysterectomy is performed.

POP and SUI coexist in up to 80% of women with pelvic floor dysfunction (Bai et al., 2002). While these conditions are often concurrent, one may be mild or occult, which makes selection of the optimal surgical procedure challenging. The 2018 Cochrane review suggested that there was insufficient evidence on whether sacrocolpexy with concurrent surgery for SUI improves urine leakage after surgery (Baessler et al., 2018). Preoperative urinary stress testing is needed to detect potential occult SUI.

With the recent FDA banning of transvaginal meshes, it is imperative to plan a meshless era, with alternatives to POP repair. The emergence of alternatives to sacrocolpopexy widens the panel of POP management possibilities with potential benefits. Alternative techniques are not widely used but the development of mesh-less laparoscopic treatments of prolapse may avoid some of graft- related complications. In addition, in some cases when access to the promontory is challenging (obese patients, unusual low position of the iliac vessels), pectopexy and LS by laparoscopy could avoid difficult dissection of this area.

## Conclusions

Considering the results of this survey, we note a lack of standardisation of many surgical steps of LSCP. In the current literature, there is a lack of overwhelming evidence across all of the surgical steps, thus making it very difficult to standardise recommendations. Furthermore, we recognise that the treatment of POP patients is usually an individualised approach depending on anatomy and presenting symptoms. To optimise efficiency in every situation, there is a need to disseminate the knowledge of alternative techniques (LS, LP and native tissue repair), which are currently still not widely used. At the same time, large randomised clinical trials evaluating any of these alternative techniques should be conducted to compare functional and anatomical results to the gold standard sacrocolpopexy. Furthermore, for laparoscopic POP repair, it is of utmost importance to have robust suturing skills and dissection competencies. The learning process to perform the surgical repair should be well organised, in specific endoscopic training courses.

## Appendix: scan QR

https://qrco.de/beLezn

**Figure:**